# Clinical, surgical characteristics and long-term outcomes of lumbar hernia

**DOI:** 10.1186/s12893-021-01328-7

**Published:** 2021-08-26

**Authors:** Chaoyong Shen, Guixiang Zhang, Sen Zhang, Yuan Yin, Bo Zhang, Yinghan Song, Wenzhang Lei

**Affiliations:** 1 Department of Gastrointestinal Surgery, West China Hospital, Sichuan University, Chengdu, 610041 Sichuan China; 2 Department of Gastrointestinal Surgery, Chengdu Second People’s Hospital, Chengdu, 610041 Sichuan China; 3 Department of Day Surgery Center, West China Hospital, Sichuan University, Chengdu, 610041 Sichuan China

**Keywords:** Lumbar hernia, Mesh repair, Surgery, Anaesthesia

## Abstract

**Background/Aim:**

Lumbar hernia is caused by a defect in the abdominal wall. Due to its rarity, there is no established consensus on optimal treatment for lumbar hernia yet. Thus, we here investigated the clinical, surgical characteristics and outcomes of lumbar hernia by collecting 28 such patients from our hospital.

**Methods:**

Patients diagnosed with lumbar hernia from our institution between April 2011 and August 2020 were retrospectively collected in this study. Demographics, clinical characteristics and surgical information were recorded.

**Results:**

A consecutive series of 28 patients with lumbar hernia were retrospectively collected, including 13 males (46%) and 15 females (54%). The ages of the patients ranged from 5 to 79 years (median: 55 years), with a mean age of 55.6 ± 14.9 years. A total of 7 cases had a history of previous lumbar trauma or surgery. There were 11 (39%), 15 (54%) and 2 (7.1%) cases had right, left and bilateral lumbar hernia, respectively. Superior and inferior lumbar hernia were found in 25 (89%) and 3 (11%) patients. General anesthesia was adopted in 16 cases (group A), whereas 12 patients received local anesthesia (group B). Patients in the group B had a shorter hospital stay than that of the group A (3.5 ± 1.3 days vs. 7.1 ± 3.2 days, *p* = 0.001), as well as total hospitalization expenses between the two groups (2989 ± 1269 dollars vs. 1299 ± 229 dollars, *p* < 0.001). With a median follow-up duration of 45.9 months (range: 1–113 months), only 1 (3%) lumbar hernias recurred for the entire cohort.

**Conclusions:**

Lumbar hernia is a relatively rare entity, and inferior lumbar hernia is rarer. It is feasible to repair lumbar hernia under local anesthesia.

## Introduction

The lumbar hernia, is defined as the protrusion of an organ (either intraperitoneal or extraperitoneal) or extraperitoneal contents through a defect in the posterolateral abdominal wall [1], which was first proposed in 1672 by Barbette and the first true case was published by deGarangeor in 1731 [2, 3]. The lumbar region is surgically defined as space between the twelfth rib superiorly, the iliac crest inferiorly, the erector spinae medially, and the external oblique laterally; anatomically, lumbar hernias can be categorised as superior (Grynfeltt-Lesshaft triangle) and inferior (Petit triangle) lumbar hernia [4]. Because the clinical manifestations are often vague or asymptomatic, the diagnosis of lumbar hernia is difficult and is usually not suspected initially. Low suspicion may lead to delayed diagnosis or misdiagnosis of other soft tissue lesions, such as subcutaneous lipoma, retroperitoneal tumor, abscesses, fibromas or perirenal abscess [5, 6]. Normally, surgical treatment of lumbar hernias is essential because of risks of incarceration, strangulation and perforation [7–9]. However, surgical repair can be often difficult considering the location of the hernia and the surrounding bony structures [1, 10].

Previously, with only a few hundred of patients reported, lumbar hernias are extremely rare [9, 11]. In view of the sparsity of lumbar hernia, a hernia surgeon may only come across one case throughout their career [11]. Up to now, there is little information about the clinical features, surgical treatment and postoperative follow-up of lumbar hernia. There is still ongoing discussion regarding which is the optimal surgical technique to be employed for lumbar hernias [9]. Therefore, we investigated the clinical features, treatments, and long-term follow-up outcomes of lumbar hernias based on data obtained from 28 consecutive patients at our institution in the present study.

## Materials and methods

### Patients selection

All patients diagnosed with lumbar hernia from our institution between April 2011 and August 2020 were retrospectively recruited in this study. Patients with incomplete medical records or without operation were excluded. Abdominal computed tomography and/or ultrasonography were routinely performed preoperatively for each patient. All data were obtained from the electronic medical chart, including patient’s age, sex, side of hernia, previous history of lumbar surgery or trauma, anesthesia methods, body mass index (BMI), surgery-related information, total hospitalization expenses and co-morbitity, etc. Written informed consent were obtained from each patient in this cohort. This study was approved by the Institutional Review Board of West China Hospital and was carried out in accordance with the declaration of Helsinki.

### Anaesthetic and surgical procedure

General or local infiltration anesthesia was used for tension-free lumbar hernia mesh repair in this study. No sedation or analgesia was preoperatively used as premedication for those who under local infiltration anaesthesia. The local anesthetics solutions were comprised of 20 ml of 2% lidocaine, 10 ml of 1% ropivacaine and 2 ml of 0.1% epinephrine, and adding normal saline to the total amount of 160 ml. Finally, the concentration of lidocaine and ropivacaine was 0.25% and 0.06% respectively. Stepwise infiltration anaesthesia was performed using a 10-ml syringe and a 22-gauge needle. In general, 40–50 ml were injected for unilateral lumbar hernia. Additionally, the patients who under general anesthesia were given the following drugs: inhalation anesthetics, propofol, sufentanil, atracurium, penehyclidine, midazolam, analgesics and antiemetics.

**Fig. 1:**
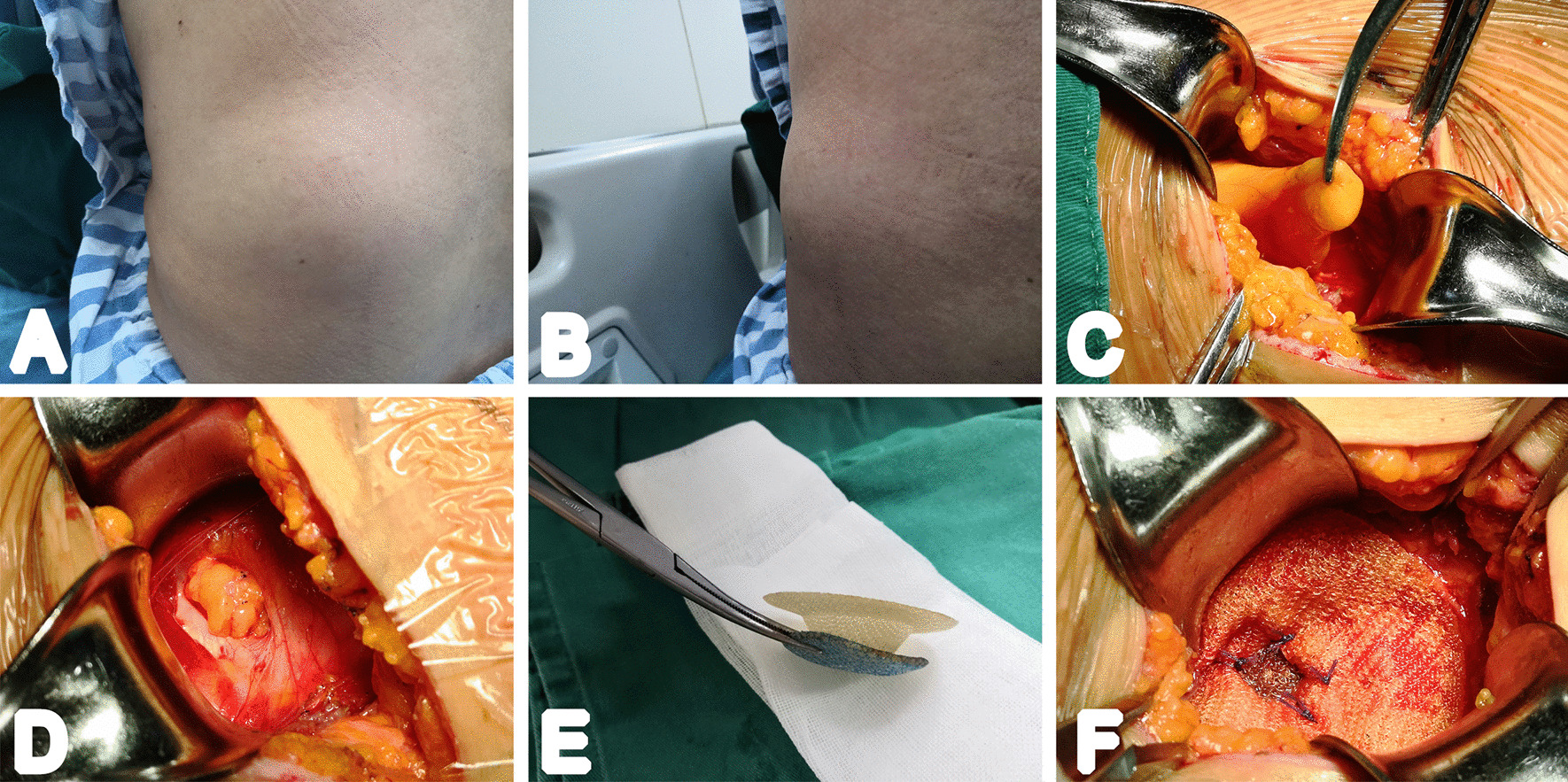
Patients underwent lumbar hernia repair with UPP under local anesthesia. **A** and **B** lumbar hernia anterior and lateral view; **C** fully free exposure of hernia sac; **D** the hernia sac was reduced; **E** appearance of mesh device; **F** the exposed hernia defect with the mesh placed

After anesthesia, a transverse incision in the flank directly over the hernia was made for most patients according to the location and size of hernia sac. Stepwise subcutaneous dissection and blunt dissociation of muscles (some overlying stretched muscle fibers were resected if necessary to expose the defect) were used to expose the hernia sac. And then, the hernia sac was dissected from its surroundings and reduced. A pre-peritoneal plane was created with blunt swab dissection. In the present study, mesh repairs were made using the ULTRAPRO™ PLUG (UPP, Ethicon, Norderstedt, Germany), ULTRAPRO™ Hernia System (UHS, Ethicon, Norderstedt, Germany), and PROCEED™ Surgical Mesh (PROCEED, Ethicon, Somerville, USA) according to the size and location of abdominal wall defect. After reducing the sac (especially for those with small hernia defect), the anchor of the UPP was then placed through the defect into the preperitoneal space without any suturing, as it would unfold automatically due to its elasticity. The rim was then sutured onto the margins of the defect with 3–0 absorbable suture (Fig. 1). For the relatively large hernia ring, after the hernia sac was fully reduced and the preperitoneal space was separated, the bottom mesh of UHS device was inserted through the defect; during the placement of mesh, it is essential to ensure that the bottom mesh was extended 2–3 cm or more from the defect edge, and the upper mesh was then sutured with the defect surface. Additionally, some patients with large abdominal wall defect, transabdominal surgical approach was performed and the PROCEED mesh was used. If hernia sac was huge, it was excised intraoperatively. The mesh with the appropriate size would be placed according to the defect of the abdominal wall (mesh edge beyond defect range at least 5 cm), and the mesh was flattened and fixed properly. And then, the wound was closed. The drainage tube was not placed routinely unless the wound was large.

### Follow-up and statistical analysis

All patients were followed up by telephone calls and outpatient clinic visits, and the last follow-up time was September 2020. Calculations statistical analysis was performed with the Statistical Package for the Social Science (SPSS) version 21.0 for Windows (SPSS Inc, Chicago, IL, USA). Continuous variables were expressed as mean ± standard deviation or median (range). Measurement data was analyzed by variance analysis. Categorical were described as frequencies and percentage, and compared with Chi-square or Fisher’s exact test. All *p* values were two-sided, with *p* < 0.05 indicated statistically significant.

## Results

### Patient and clinical characteristics

**Fig. 2:**
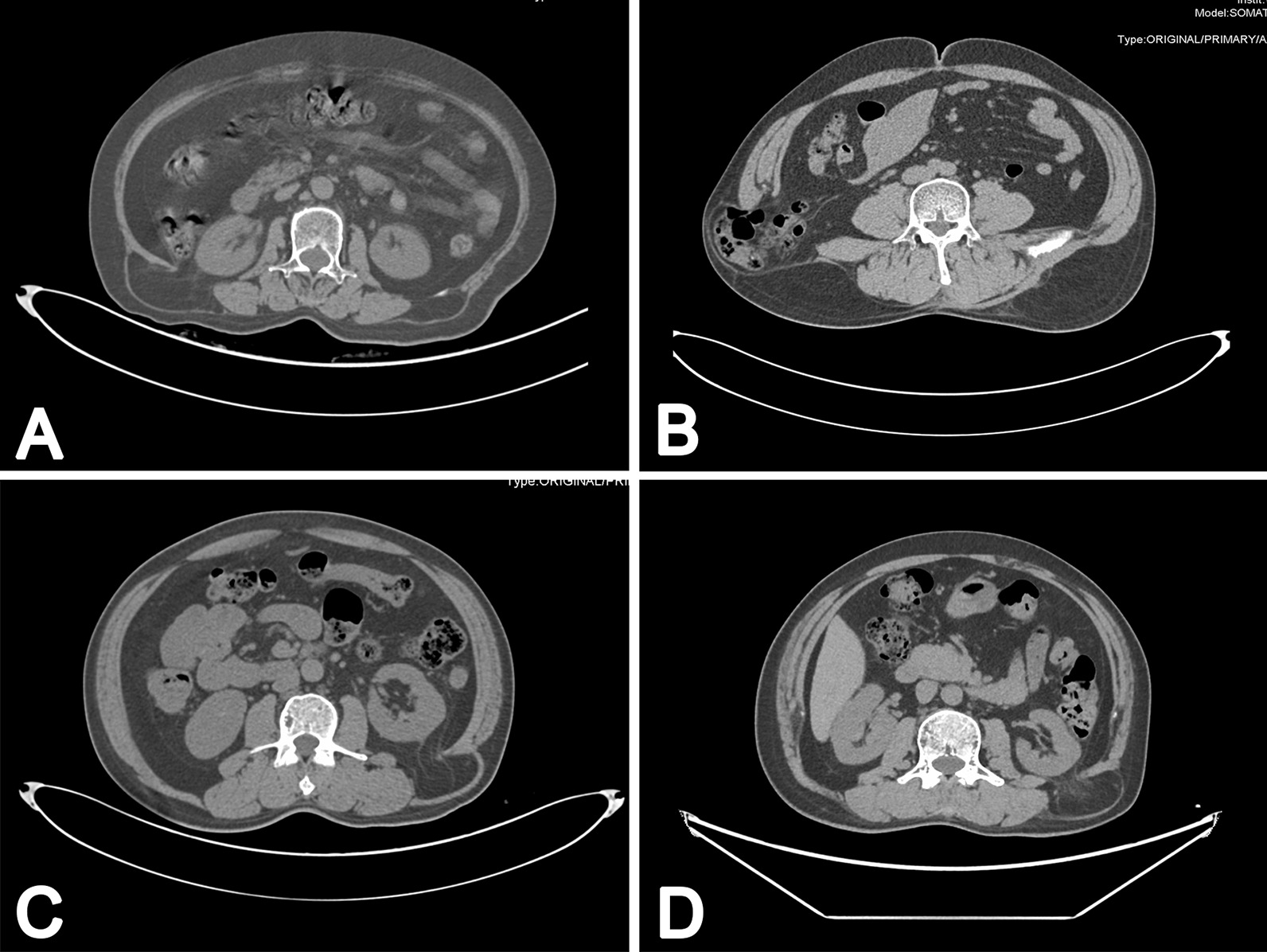
Abdominal CT showing the hernia content. **A** the bilateral lumbar hernia was showed; **B** CT demonstrating herniation of part of ascending colon bowel through a right abdominal wall defect; **C** and **D** CT showing local abdominal fat herniation into subcutaneous fat layer in the left lumbar back

Until August 2020, a consecutive series of 28 patients with lumbar hernia in our institution were retrospectively collected, including 13 males (46.4%) and 15 females (53.6%), with the male-to-female ration of 0.87 (Table 1). The ages of the patients ranged from 5 to 79 years (median 55 years), with a mean age of 55.6 ± 14.9 years. Almost all patients presented with a history of a painless mass in the lumbar region. A total of 7 cases had a previous history of lumbar trauma (one case) or surgery. Only 1 5-year-old patient had congenital lumbar hernia (unilateral), while the remaining patients (27 cases) had acquired lumbar hernia; of the 27 patients, 20 (71.4%) cases were primary, while a total of 7 (25.0) patients were secondary. There were 11 (39.3%), 15 (53.6%) and 2 (7.1%) cases had right, left and bilateral lumbar hernia for the entire cohort, respectively. In other words, there were 30 lumbar hernias in this study. Superior and inferior lumbar hernia were found in 25 (89.3%) and 3 (10.7%) patients; the four hernia sacs of 2 patients who diagnosed with bilateral lumbar hernia were all protruded through the superior lumbar triangles. Moreover, two abdominal wall defects were intraoperatively observed in 1 patient with unilateral lumbar hernia. Protrusion of intraperitoneal content (colon) was observed in 2 cases, but no incarceration or strangulation was found (Fig. 2). Incarceration was found in 13% (4/30) of lumbar hernias, but no strangulation occurred for the entire cohort. Moreover, a total of 2 patients were complicated with inguinal hernia, one of which had bilateral inguinal hernia. There were 16 and 12 patients underwent general and local anesthesia, respectively.

**Table 1:** Demographic and clinical characteristics of lumbar hernia (n = 28)

Parameters	n (%)
Sex	
Male	13 (46)
Female	15 (54)
Age (year: median [range])	55 (5–79)
Previous lumbar trauma or surgery	7 (25)
BMI (kg/m^2^, mean ± SD)	23.0 ± 3.4
Congenital/acquried lumbar hernia	1 (4)/27 (96)
History of COPD	2 (7)
Side of lumbar hernia	
Right	11 (39)
Left	15 (54)
Bilateral	2 (7.1)
Surperior/inferior lumbar hernia	25 (89)/3 (11)
Co-morbitity^‡^	
Present	8 (29)
Absent	20 (71)
Combined with inguinal hernia	2 (7.1)
Size of abdominal wall defect (cm, mean ± SD)	3.2 ± 1.84
Anesthesia method	
General	16 (57)
Local	12 (43)
Hospital stay (days, mean ± SD)	5.5 ± 3.1

*BMI* Body Mass Index, *SD* standard deviation, *COPD* chronic obstructive pulmonary disease

^‡^Includes diabetes mellitus, chronic cardiovascular disease and liver cirrhosis

### Surgical outcomes

All patients underwent classical open repair. General anesthesia was applied in 16 cases (group A), whereas 12 patients received local anesthesia (group B). A total of 27 patients underwent mesh repair, and primary closure was performed in one case with congenital lumbar hernia. Totally, 25 cases underwent extraperitoneal repair. No patient in the group B required conversion to general or spinal anaesthesia. There were no significant between-group differences in sex, age, BMI, and side of lumbar hernia (*p* > 0.05). Though a trend for smaller size of abdominal wall defect and shorter operation time were observed in the group B when compared with group A, the differences were not significant (*p* > 0.05). No postoperative bleeding and infection occurred. Of note, patients in the group B had a shorter hospital stay than that of group A (3.5 ± 1.3 days vs. 7.1 ± 3.2 days, *p* = 0.001), as well as total hospitalization expenses between the two groups (2988.6 ± 1268.8 $ vs. 1299.0 ± 229.3 $, *p* < 0.001). With a median follow-up duration of 45.9 months (range 1–113 months), only 1 (3.3%) lumbar hernias recurred for the entire cohort. In addition, there was no significant difference with respect to chronic wound pain and foreign body sensation between the two groups during the follow-up period (Table 2).

**Table 2:** Comparison of clinical features and operation-related information between general (n = 16, group A) and local (n = 12, group B) anesthesia

	Group A	Group B	*p*
Sex (%)			0.229
Male	9 (56)	4 (33)	
Female	7 (44)	8 (67)	
Age (years)	55.4 ± 18.1	56.0 ± 9.7	0.915
BMI (kg/m^2^)	23.8 ± 4.0	22.0 ± 2.4	0.178
Side of lumbar hernia (%)			0.107
Right	8 (50)	3 (25)	
Left	6 (38)	9 (75)	
Bilateral	2 (13)	0 (0)	
Size of abdominal wall defect (cm)	3.8 ± 2.1	2.5 ± 1.2	0.069
Operation time (min)	40.0 ± 14.3	34.1 ± 5.4	0.185
Postoperative bleeding (Y/N)	0/16	0/12	–
Wound infection (Y/N)	0/16	0/12	–
Chronic wound pain (Y/N)	2/14	1/11	1.000
Foreign body sensation (Y/N)	3/13	2/9	1.000
Postoperative recurrence (Y/N)	1/15	0/12	1.000
Hospital stay (days)	7.1 ± 3.2	3.5 ± 1.3	0.001
Total hospitalization expenses ($)	2989 ± 1269	1299 ± 229	< 0.001

*BMI* Body Mass Index, *Y* yes, *N* no, *$* dollars

## Discussion

Lumbar hernias can be classified based on location and etiology [1]. According to the anatomical location of the defect, lumbar hernias were divided into Grynfeltt hernia (the superior triangle) and Petit hernia (the inferior triangle). However, blunt abdominal trauma may also create lumbar hernia, which was classified as the “diffuse” type and was not be confined to these two triangles [12, 13]. The superior lumbar triangle is an inverted triangle whose base is formed by the 12th rib and the serratus posterior inferior muscle, while the inferior lumbar triangle is an upright triangle whose base is formed by the iliac crests. The most common site for the occurrence of lumbar hernias is in the superior lumbar triangle [10, 14]. Superior and inferior lumbar hernia were found in 25 (89.3%) and 3 (10.7%) patients in the present study, which is consistent with their reports. Moreover, lumbar hernias can be divided into two categories: congenital or acquired. In all, approximately 20% of lumbar hernias are congenital [1], and acquired lumbar hernias account for 80% of lumbar hernias [13]. Congenital lumbar hernia occurs in infancy, and may be associated with musculoskeletal or other birth defects [10, 15–17]. Furthermore, acquired lumbar hernias can be further classified as either primary or secondary. The former type (spontaneous) is precipitated by conditions associated with increased intra-abdominal pressure or aging, chronic bronchitis, and extreme thinness, etc. Secondary-type lumbar hernias, are often associated with surgical incisions, trauma, or lumbar abscess, which are estimated to represent 25% of lumbar hernias [1, 18]. Normally, after flank incisions usually for retroperitoneal operations as in urology or when harvesting a bone graft from the iliac crest, some patients are more likely to suffer from lumbar hernia. Consistent with previous reports, our study also found that some patients had a history of lumbar trauma and surgery.

The diagnosis of lumbar hernia is often difficult and is not suspected initially. Firstly, clinical presentation for lumbar hernias is asymptomatic or variable. Patients may present with flank pain, back or abdominal discomfort and painless mass. In addition, the challenge in diagnosis also stems from a lack of awareness and insufficient cases. Physical examination may reveal a reducible mass that may increase in size with coughing and Valsalva maneuver [5]. A reducible mass with cough impulse, however, may not always be present due to small defects, obesity or other factors. Computed tomography (CT) is exceedingly useful in the diagnosis of lumbar hernias as it can delineate the location and size of the defect, as well as delineate the muscular and fascial layers and the contents within the hernia sac, so as to provide the basis for making a reasonable treatment plan [19, 20]. Previous study has shown that abdominal CT scanning was used in 56 of 66 instances and was 98% sensitive for diagnosis of traumatic lumbar hernias [13]. Moreover, CT can also effectively rule out the other differential diagnoses of lumbar hernias, such as lipomas, abscesses, and retroperitoneal tumors [10, 18].

Lumbar hernias are more often found on the left side and in the upper lumbar triangle [9]. In the present study, we also found that a majority of lumbar hernias located in the left and in the superior triangle. There were 2 (7.1%) patients having coexisting inguinal hernia in this study, which was lower than that of reported data [8]. Moreover, bilateral lumbar hernias are even less frequently documented, and most of the reports are case reports so far [15, 21]. Our results have shown that there were 2 patients with bilateral lumbar hernia who underwent surgery under general anesthesia, and all hernia sacs protruded through the superior lumbar triangles. The contents of lumbar hernia may be extraperitoneal of intraperitoneal, such as extraperitional fat, colon, spleen, liver etc.; whereas, in the traumatic lumbar hernia, fat (42%), colon (41%), and small bowel (32%) were the most common hernia contents [13].

Most lumbar hernias have a propensity to undergo slow benign expansion in size over time. Once the size of defect increases, the difficulty of subsequent surgery will be increased accordingly [22]. Additionally, the reported risk of incarceration from lumbar hernias was approximately 25–30.8% [1, 9] and there was an 8% chance of strangulation [23]. In the present study, a total of 13% of lumbar hernias had incarceration but no strangulation occurred. It is likely to be related to the improvement of patients' awareness of timely medical treatment. In addition, it is recommended that these hernias should not be managed conservatively without surgery [17, 22]. Surgical repair to eliminate the defect, reconstruct and strengthen the abdominal wall may be the most effective treatment for lumbar hernias. Hence, surgical treatment with either open or laparoscopic is both the treatment of choice. Recently, successful laparoscopic repairs of lumbar hernia defects have been reported [24]. In laparoscopic repair, the main advantage is that it seems to ensure the proper placement of mesh, and also it has been shown to be more favorable surgical outcomes (shorter operating time and shorter hospital stay, etc.) than open repair. However, open repair is the most commonly used technique for lumbar hernias currently [6]. In the present study, all patients underwent open surgery. The hernia can be repaired through a transabdominal or extraperitoneal approach. Generally, repair technique largely depend on the size of hernia and available facilities. Primary closure with interrupted tension-free sutures for lumbodorsal fascia has the potential to be effective in small hernias, but sometimes the failure rate is also high [13]. For large hernias, they can be repaired by using non-absorbable prosthetic material [1, 10]. In this study, a total of 27 patients underwent mesh repair, and primary closure was performed in one case with congenital lumbar hernia. On the whole, with limited cases to compare surgical approaches and surgical techniques, the ideal surgical treatment is inconclusive yet.

Currently, there is no relevant study to explore the feasibility and safety in the treatment of lumbar hernia under local anesthesia. In the present study, the hospital stays for the local anesthesia are significantly less when compared to the general anesthesia, as well as the total hospitalization expenses. However, further explorations using a large sample are warranted. The long-term follow-up and recurrence data are scanty. van Steensel et al. reported that the 2.0% had a recurrence after surgical repair for primary lumbar hernia [9]. However, they have pointed out that an underestimation of the recurrence rates may be occurred due to publication bias. By comparison, the recurrence rate was 1 out of 30 (3.3%) hernias in this study, which is higher than that of their data. According to the literature, predictors associated with an increased likelihood for recurrence of lumbar hernias are those with diffuse ones and a defect size larger than 16 cm [24].

However, our study had several limitations. Due to the nature of the retrospective study, we can not draw a convincing conclusion; van Steense et al. have reported that 2.0% had a recurrence after surgical repair for lumbar hernia [9]. With a median follow-up duration of 45.9 months, only 3% lumbar hernias recurred for the entire cohort, which is in line with their results. However, due to the small sample size of our study, our data may not reflect real recurrence rates, which is also the chief criticism of our study. As such, multicenter prospective researches are warranted in the near future.

## Conclusions

In summary, lumbar hernia is a relatively rare entity, and inferior lumbar hernia is rarer. There are currently no guidelines for the ideal method of repair. It is feasible to repair lumbar hernia under local anesthesia.

## Data Availability

The data will not be made available in order to protect the participant’s identity. Those interested parties can contact Prof. Zhang (hxwcwk@126.com) who will provide the whole raw data.

## Abbreviations

BMI: Body Mass Index
SD: Standard deviation
COPD: Chronic obstructive pulmonary disease
CT: Computed tomography
